# Underuse of primary healthcare in France during the COVID-19 epidemic in 2020 according to individual characteristics: a national observational study

**DOI:** 10.1186/s12875-022-01792-x

**Published:** 2022-08-09

**Authors:** Philippe Tuppin, Thomas Lesuffleur, Panayotis Constantinou, Alice Atramont, Carole Coatsaliou, Emilie Ferrat, Florence Canouï-Poitrine, Gonzague Debeugny, Antoine Rachas

**Affiliations:** 1 Direction de la Stratégie, des Etudes et des Statistiques, Caisse Nationale de L’Assurance Maladie, 26-50, avenue du Professeur André Lemierre, 75986 Paris Cedex 20, France; 2 Université Paris Est Créteil, IMRB, INSERM, 94000 Créteil, France; 3 Université Paris-Est Créteil, Département Universitaire d’Enseignement en Médecine Générale, Créteil, France; 4 Service de Santé Publique, APHP, Hôpital Henri-Mondor, Créteil, France

**Keywords:** Ambulatory healthcare use, Chronic diseases, COVID-19, Deaths, Epidemic trends, France, Lockdown, Observational study

## Abstract

**Background:**

The organization of healthcare systems changed significantly during the COVID-19 pandemic. The impact on the use of primary care during various key periods in 2020 has been little studied.

**Methods:**

Using individual data from the national health database, we compared the numbers of people with at least one consultation, deaths, the total number of consultations for the population of mainland France (64.3 million) and the mean number of consultations per person (differentiating between teleconsultations and consultations in person) between 2019 and 2020. We performed analyses by week, by lockdown period (March 17 to May 10, and October 30 to December 14 [less strict]), and for the entire year. Analyses were stratified for age, sex, deprivation index, epidemic level, and disease.

**Results:**

During the first lockdown, 26% of the population consulted a general practitioner (GP) at least once (-34% relative to 2019), 7.4% consulted a nurse (-28%), 1.6% a physiotherapist (-80%), and 5% a dentist (-95%). For specialists, consultations were down 82% for ophthalmologists and 37% for psychiatrists. The deficit was smaller for specialties making significant use of teleconsultations. During the second lockdown, the number of consultations was close to that in 2019, except for GPs (-7%), pediatricians (-8%), and nurses (+ 39%). Nurses had already seen a smaller increase in weekly consultations during the summer, following their authorization to perform COVID-19 screening tests. The decrease in the annual number of consultations was largest for dentists (-17%), physiotherapists (-14%), and many specialists (approximately 10%). The mean number of consultations per person was slightly lower for the various specialties, particularly for nurses (15.1 vs. 18.6). The decrease in the number of consultations was largest for children and adolescents (GPs: -10%, dentists: -13%). A smaller decrease was observed for patients with chronic diseases and with increasing age. There were 9% excess deaths, mostly in individuals over 60 years of age.

**Conclusions:**

There was a marked decrease in primary care consultations in France, especially during the first lockdown, despite strong teleconsultation activity, with differences according to age and healthcare profession. The impact of this decrease in care on morbidity and mortality merits further investigation.

## Background

The COVID-19 epidemic and the measures taken to limit its spread, such as lockdowns, curfews, and the tightening of sanitary rules, greatly affected the functioning of healthcare systems. This context led to shortages of medical equipment, and to hospital capacity being exceeded, particularly for emergency departments and intensive care units, with care reorganized to prioritize the management of patients with COVID-19, leading to a decrease in hospital consultations and a possible increase in deaths in non-hospitalized individuals [1–8].

Advanced age is one of the main risk factors for severe disease and death from COVID-19, but chronic diseases, such as cardiovascular or kidney disease, diabetes, hypertension, and obesity, are also important risk factors, as is social deprivation [9, 10]. Patients with chronic diseases requiring continuity of care to prevent a worsening of the condition or the occurrence of complications, were faced with the postponement or cancellations of consultations, hospital stays, and scheduled surgeries [11–13]. In addition, some individuals feared becoming infected or contributing to the overcrowding of healthcare facilities, which limited their excursions from home and their use of healthcare [13, 14]. Most of the many studies performed during this period focused on particular diseases or care sectors, such as hospitals. Only a few studies have examined the impact on the use of primary care specialties, by type of insurance, epidemic level in the region concerned, or through questionnaires [15–21].

France (67.4 million inhabitants) was greatly affected by the two waves of the epidemic that hit in 2020. Almost 280,000 hospitalizations for COVID-19 were recorded from March 19 until the end of the year in 2020 [22]. There were 66,000 recorded deaths from COVID-19 in hospitals and care homes [22]. Almost 670,000 deaths from all causes were recorded in 2020, a figure 9% higher than that for 2019 [23]. A first lockdown of almost eight weeks was implemented in the spring of 2020 and a second, slightly shorter, in the fall. During the first lockdown, the population was instructed to restrict movement outside the home to the minimum necessary. Companies were instructed to make maximum use of working from home, and schools, shops, and “non-essential” businesses, social and leisure establishments were closed, with infractions of the rules punished. The second confinement was less strict: schools remained open, and the activity of many professional sectors was maintained.

The primary objective of this study was to describe the change in healthcare use and consultations for the various primary care, medical, and paramedical professionals for the French population for the year 2020, and any decreases relative to 2019. This analysis was broken down by sociodemographic characteristics, chronic diseases identified in 2019, and the rate of hospitalization for COVID-19 in the area (*département*, a French administrative unit similar in size to a county) of residence.

## Methods

### The national health database (Système national de données de santé—SNDS)

France provides universal medical coverage for all residents. Those insured can choose their own healthcare professionals, although they pay a small financial penalty if they consult certain specialists without referral from their declared personal physician (generally their GP). The doctors are responsible for prescribing nursing care and physiotherapy. A national nomenclature is used to identify the specialty of the healthcare professional and the consultations or procedures reimbursed. The SNDS contains an exhaustive collection of the characteristics of insured individuals legally residing in France, together with their consultations, medical acts, and prescriptions covered or reimbursed by the health insurance system, together with the corresponding dates [24]. It contains no clinical or paraclinical information from primary care consultations. A pseudonymized identifier is used to link all of this information to that in databases for stays in public and private hospitals, including the diagnostic codes associated with the stay. The SNDS also contains information about long-term disease status (*affection de longue durée*, ALD) for many chronic diseases. This status is assigned in response to a request from the patient’s doctor and allows a higher level of reimbursement (100%). ALDs and hospital diagnoses are coded according to the International Classification of Diseases version 10 (ICD 10). Deaths are updated over time, with data from civil status registries and collected by the National Institute for Statistics and Economic Studies (*l'Institut National de la Statistique et des Études Économiques*—INSEE). Cause of death data are available from the SNDS, but not until two to three years after the event; such data could not, therefore, be included in this study.

### Participants

All those insured in 2019 or 2020 were selected from the SNDS, except for those residing in overseas territories (DROM, 2.7 million) or those with no outpatient or hospital care reimbursed in 2019 (almost 0.4 million people). The DROMs were excluded because the COVID-19 epidemic varied in intensity in a manner different from that in mainland France, resulting in differences in the dates of confinement periods and curfew conditions relative to mainland France. We thus included 64.3 million individuals, with a mean age of 41.9 years (Standard deviation ± 24.5 years) in 2020.

### Outcomes

The generic term “consultation” encompasses reimbursement for classical consultations, teleconsultations, home visits, or at least one medical or paramedical act on a given day, including consultations at a healthcare center. The consultations concerned were performed by primary healthcare professionals: general practitioners, dentists, nurses, physiotherapists, midwives, and certain specialists with significant outpatient activity (ophthalmologists, gynecologists, dermatologists, cardiologists, ENT specialists, gastroenterologists, rheumatologists, pediatricians, pulmonologists, psychiatrists, and endocrinologists). In France, any doctor can offer a teleconsultation, whatever his specialty and practice sector. Its proposal is the sole decision of the doctor who must judge the relevance of a medical treatment in teleconsultation rather than during a traditional face-to-face consultation. The patient's consent is required. In order to ensure the best quality and safety of care, regular follow-up must be done by alternating face-to-face consultation and teleconsultation. The use of teleconsultation is based on a territorial logic. The doctor who performs a teleconsultation must be located close to the patient's home and thus make it possible to organize a face-to-face consultation if, at the end of the teleconsultation, this proves necessary.

### Covariates

The sociodemographic characteristics considered were age, sex, and social deprivation estimated with a geographic index corresponding to the town of residence and broken down into quintiles [25]. This index was constructed from data provided by the INSEE: median fiscal income per consumption unit, the percentage of high-school graduates aged 15 and over in the population, the percentages of manual workers and unemployed individuals in the working population (15–64 years of age). The intensity of the epidemic during the first confinement and for the entire year of 2020 was estimated by the crude rate of new hospitalizations for COVID-19 (per 100,000 inhabitants) in the area of residence, provided by Public Health France, and broken down into quartiles.

Comorbid conditions were identified with the healthcare expenditures and conditions mapping tool for the year 2019. Algorithms were developed for the identification of 58 non-exclusive health conditions (grouped into 15 categories) from the medical information available in the SNDS. These algorithms were based on the following elements: LTD ICD-10 codes, ICD-10 codes of diagnoses related to hospitalizations during the year studied (or up to five years prior to hospitalization, depending on the algorithm), drugs specific to certain chronic diseases, and, for several diseases, laboratory tests, medical procedures, lump sums, and diagnosis-related groups [26]. The severity of the patient’s state of health was assessed with the Mortality-Related Morbidity Index (MRMI), which predicts two-year mortality in insured individuals aged 65 years and over [26].

### Periods studied

The year 2020 was studied globally, week-by-week, and, more specifically, during the two lockdowns. The first lockdown was announced on March 12 and lasted from March 17 to May 10. A gradual easing of lockdown conditions then occurred, from May 10 to June 22. After the summer, restrictions on gatherings and the closure of bars and restaurants were gradually implemented from September 26 (week 39), culminating in a national curfew beginning on October 14, followed by the announcement of a second lockdown on October 28, which lasted from October 30 to December 14.

### Data analyses

The number of individuals with at least one consultation was calculated for each healthcare profession and for the similar period of 2019 and 2020 (weekly, confinement, whole year). We also determined the number of consultations and their proportions, to estimate differences in activity between the two years. Those from 2020 have been reported. For the two years, only full weeks were included, 51 in total, excluding a few days at the start and end of the year. Week 1 was, therefore, the first full week, in both 2019 and 2020. The mean number of consultations was calculated for individuals with at least one consultation over the period studied, to estimate changes in the intensity of healthcare consumption. All-cause mortality (per 100,000 inhabitants) was calculated each year with an overall ratio between the two years for each covariate studied. Curves were generated by healthcare profession, to monitor weekly changes in the proportion of individuals with at least one consultation. Similar curves were generated to visualize the changes in the proportion of teleconsultations in 2020 among the total number of consultations for each medical profession.

Given the near-exhaustiveness of the study population and the large sample size, statistical tests were not performed [27]. SAS software was used (version 7.13, SAS Institute Inc, Cary, NC, USA) for all statistical analyses.

## Results

### Weekly differences in the number of consultations by healthcare profession

**Fig. 1:**
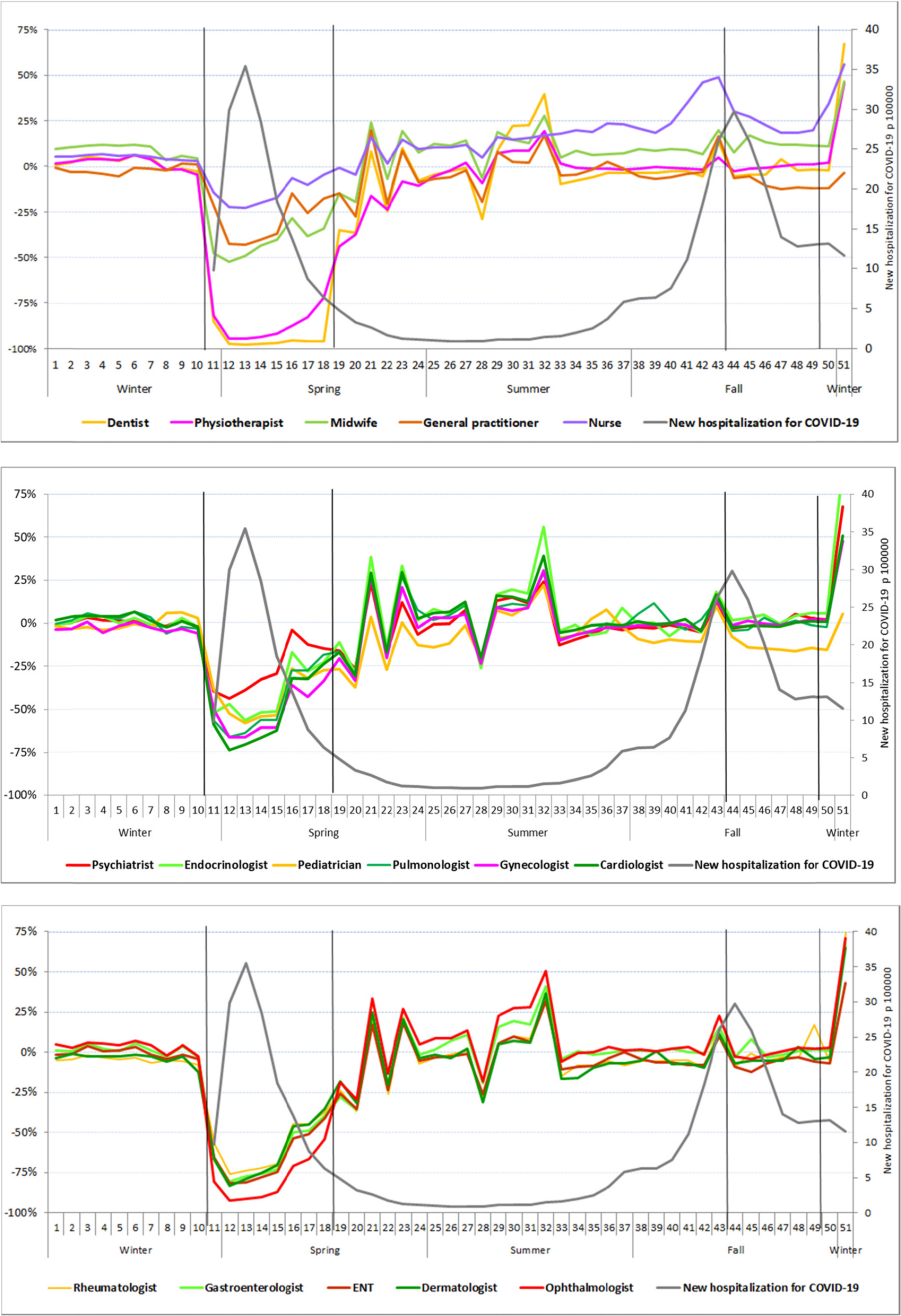
Change in the weekly number of people with at least one consultation in 2020 relative to the same week in 2019, by type of healthcare professional, for mainland France. First national lockdown W11-W18 (March 17 to May 10, 2020), Second national lockdown W44-W49 (October 30 to December 14, 2020). Weekly rates of new hospitalizations for COVID-19, used as a marker of epidemic intensity

The weekly numbers of people with at least one consultation were similar for the first seven weeks of 2019 and 2020, at about 0 to + 10%, depending on the healthcare profession considered (Fig. 1). A slight decrease was then observed in 2020, preceding a major decline from the first week of the first lockdown (week 11, 2020). This was particularly true for dentists and physiotherapists (decrease of almost 100%), midwives (-50%), specialist doctors, such as ENT specialists (-75%) and ophthalmologists (-90%). A more moderate decrease was observed for GPs, psychiatrists (-40%), and, especially, nurses (-25%). These declines remained broadly stable over the next month, and then gradually decreased in amplitude during the three weeks preceding the easing of restrictions (week 19), at different rates according to the profession considered. This dynamic continued after the end of the lockdown, with an increase, of variable speed, towards the levels of 2019. Thus, from the end of the spring to the beginning of the fall, there was almost no difference between the years, but with oscillations due to the presence of numerous public holidays during this period. However, nurses had a difference of + 20% relative to 2019, which increased further (+ 40%) before the second lockdown, subsequently returning to + 20% by the end of the year. The differences between the years for other healthcare professions also increased (from + 5 to + 20%) before the second lockdown, except for GPs (-10%) and pediatricians (-20%). Dentists, paramedics, and most medical specialists saw their ratios rise sharply in the last week of 2020.

**Fig. 2:**
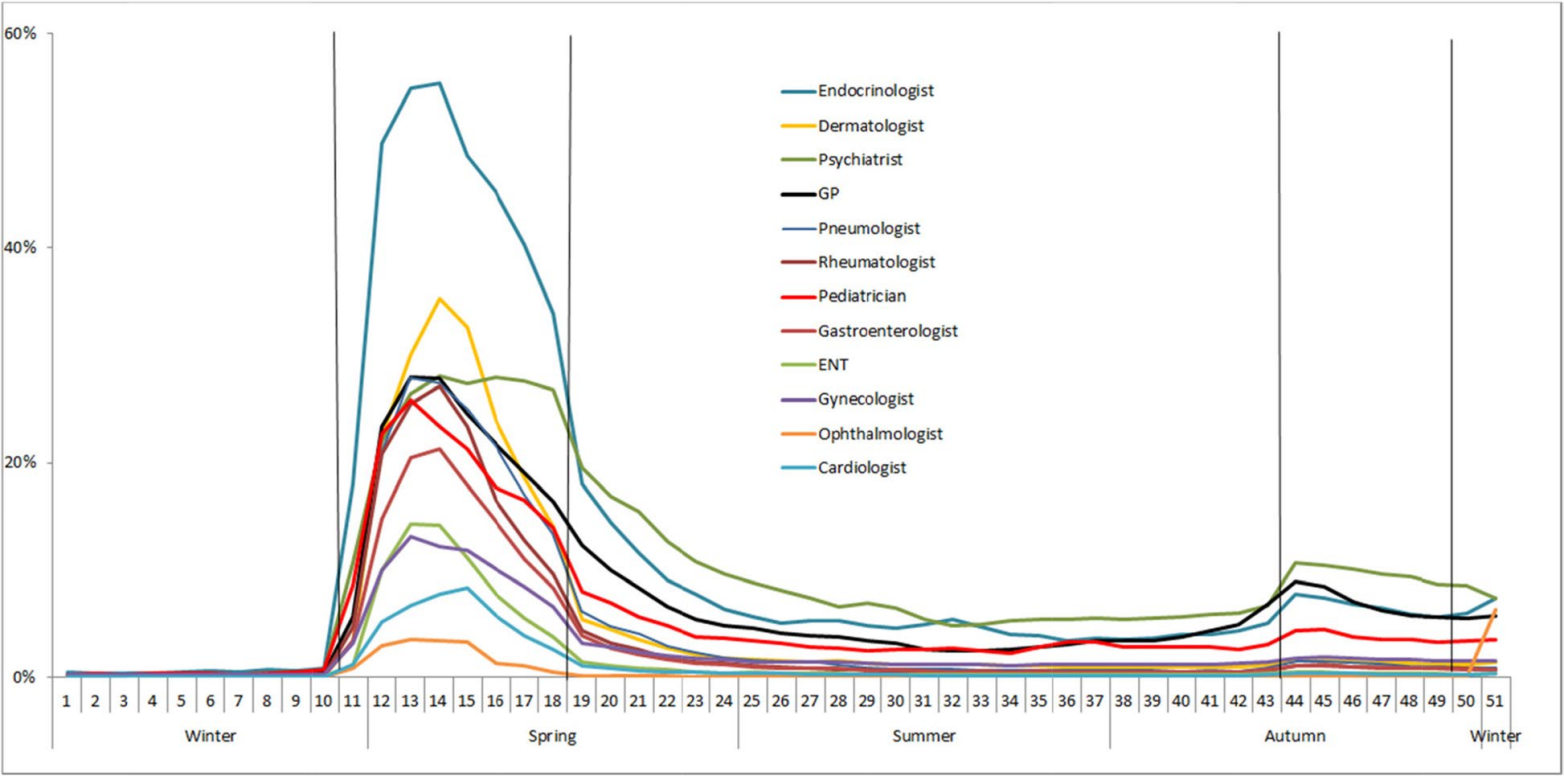
Weekly proportion of total consultations in the form of teleconsultation in mainland France in 2020, by specialty. First national lockdown W11-W18 (March 17 to May 10, 2020). Second national lockdown W44-W49 (October 30 to December 14, 2020)

Teleconsultation activity was almost null in 2019 and early 2020, but gradually increased as a proportion of all weekly consultations during the first lockdown (week 11), peaking in week 14 for endocrinologists (55%), dermatologists (35%), psychiatrists (28%), GPs (27%), and, to a lesser extent, cardiologists (8%) and ophthalmologists (3%) (Fig. 2). Teleconsultation rates rapidly decreased before the end of the first lockdown, and then stabilized to reach a low-level plateau in the summer (5% for psychiatrists, who had the largest share). There was a moderate rebound during the second lockdown for certain specialties (11% for psychiatrists, again with the largest share).

### Variations in the number of consultations during the lockdowns and for all of 2020, by healthcare profession

Overall, during the first 55-day lockdown in 2020, the number of patients who had at least one consultation fell for all professionals: nurses (-28%), GPs (-34%), midwives (-39%), physiotherapists (-80%), and dentists (-95%) (Table 1). For specialists, the decrease ranged from 37% for psychiatrists to 82% for ophthalmologists. The mean number of consultations per individual was higher in 2020 than in 2019 for nurses (11.5 vs. 8.8) and psychiatrists (3.5 vs. 3.0) and lower for physiotherapists (5.8 vs. 8.0) and dentists (1.1 vs. 1.4).

**Table 1:** Description of the use of primary healthcare during the lockdown periods throughout the whole of 2020 relative to the corresponding periods in 2019 in mainland France

	**At least one consultation 2020**			**Ratio 2020/2019**			**Total consultations ratio 2020/2019**			**Mean number of consultations per individual with at least one consultation**					
***N* = 64.3 million**	**LD 1**	**LD 2**	**2020**	**LD 1**	**LD 2**	**Year**	**LD 1**	**LD 2**	**Year**	**LD 1**		**LD 2**		**Year**	
										**2019**	**2020**	**2019**	**2020**	**2019**	**2020**
**Specialty**	%	%	%	%	%	%	%	%	%	mean	mean	mean	mean	mean	mean
Generalist	26.0	33.6	80.1	-34	-7	-3	-32	-5	-8	1.6	1.6	1.5	1.5	4.9	4.6
Nurse	7.4	15.2	41.5	-28	+ 39	+ 29	-7	+ 9	+ 5	8.8	11.5	7.1	5.6	18.6	15.1
Dentist	0.5	9.8	37.1	-95	+ 1	-12	-96	+ 2	-17	1.4	1.1	1.3	1.3	2.3	2.1
Physiotherapist	1.6	6.7	15.2	-80	-3	-8	-85	+ 6	-14	8.0	5.8	7.3	8.0	23.8	22.2
Midwife	0.5	0.9	2.9	-39	+ 19	+ 10	-44	+ 16	0	2.9	2.6	2.6	2.6	6.0	5.4
Ophthalmologist	1.0	5.4	26.7	-82	+ 4	-7	-82	+ 4	-10	1.1	1.1	1.1	1.1	1.5	1.4
Gynecologist	1.1	2.4	10.2	-59	+ 4	-9	-55	+ 5	-10	1.3	1.4	1.2	1.2	1.9	1.9
Cardiologist	1.0	2.1	9.6	-55	+ 2	-5	-55	+ 2	-8	1.4	1.4	1.3	1.3	2.0	1.9
Dermatologist	0.7	1.8	9.0	-66	-1	-12	-67	0	-14	1.1	1.1	1.1	1.1	1.5	1.5
ENT	0.5	1.2	6.3	-69	-4	-11	-69	-3	-14	1.1	1.1	1.1	1.1	1.6	1.5
Gastroenterologist	0.3	0.9	3.9	-66	+ 4	-7	-65	+ 4	-10	1.3	1.4	1.3	1.3	1.9	1.9
Pediatrician	0.7	0.9	3.6	-45	-8	-4	-46	-11	-13	1.4	1.3	1.3	1.3	2.9	2.7
Rheumatologist	0.4	0.8	3.2	-61	+ 2	-10	-62	+ 3	-14	1.3	1.2	1.2	1.2	2.1	2.0
Pulmonologist	0.3	0.6	2.8	-50	+ 3	-4	-48	+ 2	-7	1.3	1.3	1.2	1.2	1.8	1.8
Psychiatrist	0.8	1.2	2.4	-37	-1	-4	-25	+ 3	-5	3.0	3.5	2.7	2.8	10.1	10.0
Endocrinologist	0.2	0.4	1.6	-46	+ 7	-4	-44	+ 8	-5	1.1	1.2	1.1	1.1	1.9	1.9

LD 1: First national lockdown W11-W18 (March 17 to May 10, 2020)

LD 2: Second national lockdown W44-W49 (October 30 to December 14, 2020)

Reading grid: During the first lockdown, the percentage of people with at least one consultation with a general practitioner in 2020 was 26.0%, corresponding to a 34% decrease relative to 2019. There was also a 32% decrease in the overall number of consultations in 2020 relative to 2019. For those with at least one consultation during the study period, their mean number of consultations per person was 1.6 during the first lockdown in 2020

During the second lockdown (lasting 45 days), the decrease, in the number of patients who had at least one consultation was smaller (GPs: -7%, physiotherapists: -3%, pediatricians: -8%, ENT specialists: -4%) (Table 1). Conversely, there was a large increase for nurses (+ 39%) and midwives (+ 19%) and a more moderate increase for certain specialists (endocrinologists: + 7% and dentists: + 1%). By contrast to the first lockdown, the mean number of consultations per person was similar for GPs and specialists, lower for nurses (5.6 in 2020 vs. 7.1 in 2019), and higher for physiotherapists (8.0 vs. 7.3).

For the entire year of 2020, the annual proportion of patients with at least one consultation was higher than that for the preceding year for nurses (+ 29%) and midwives (+ 10%), was slightly lower for GPs (-3%), and generally lower for specialists, especially cardiologists (-5%), gynecologists (-9%), dermatologists (-12%), and ENT specialists (-11%). The total number of consultations was lower than that in 2019 for almost all healthcare professionals, with changes of -5% for psychiatrists and endocrinologists to -17% for dentists. Only nurses carried out more consultations (+ 5%). However, the mean number of consultations per individual with at least one consultation was lower (15.1 in 2020 vs. 18.6 in 2019), as it was, to a lesser extent, for GPs (4.6 vs. 4.9), physiotherapists (22.2 vs 23.8), and midwives (5.4 vs 6.0).

### Consultations according to individual sociodemographic characteristics

The first lockdown period saw the largest decline in the number of people who had at least one consultation for all professions, but this decline was strongest for the two- to six-year and seven- to 17-year age groups (GPs: -60%, nurses: -50%, physiotherapists: -89%), and decreased with age (85 years and over – MG: -23%, nurses: -7%, physiotherapists: -57%). For dentists, the decline in activity was substantial and similar, regardless of patient age (Tables 2 and 3). The decreases were of similar magnitude for both sexes. In terms of the deprivation index, the decrease in consultations was greatest for the most advantaged quintile for GPs (Q1: -38% vs. Q5: -32%) and was similar between quintiles for all other healthcare professions. During the first lockdown, the decrease in the number of nurse consultations was slightly smaller in the areas with the lowest hospitalization rates for COVID-19 (Q1: -25% vs. Q4: -31%). During the second lockdown, the decreases were generally smaller than those for the first lockdown for GPs, physiotherapists, and dentists, except for the youngest patients, for whom the number of consultations with nurses increased very strongly.

**Table 2:** Description of person characteristics with at least one consultation with GPs and nurses during the lockdown periods and the whole of 2020 relative to the corresponding periods in 2019, for mainland France

			**General practitioner**						**Nurse**						**Deaths**	
			**At least one consultation 2020**			**Ratio 2020/2019**			**At least one consultation 2020**			**Ratio 2020/2019**			**2020**	**Ratio 2020/2019**
	**Individuals**	**Age**	LD 1	LD 2	Year	LD 1	LD 2	Year	LD 1	LD 2	Year	LD 1	LD 2	Year		
	2020 Million	Mean	%	%	%	%	%	%	%	%	%	%	%	%	/100.000	%
**Total**	64.3	41.9	26.0	33.6	80.1	-34	-7	-3	7.4	15.2	41.5	-28	+ 39	+ 29	587.1	+ 9
**Age (years)**																
0–1	1.4	-	30.0	34.3	82.1	-43	-26	-7	0.6	1.4	7.4	-33	+ 64	+ 43	0.3	-19
2–6	3.7	-	13.9	21.9	78.3	-60	-35	-9	0.6	2.4	10.4	-51	+ 136	+ 67	0.2	-24
7–17	8.4	-	10.5	19.6	75.1	-60	-21	-6	1.0	6.4	20.9	-52	+ 212	+ 102	0.8	-1
18–25	5.8	-	17.5	23.1	74.9	-38	-9	-2	3.2	9.9	35.5	-36	+ 120	+ 63	1.8	-2
26–50	20.0	-	24.5	29.6	77.9	-28	-5	-3	4.8	12.2	39.1	-35	+ 82	+ 41	22.0	-1
51–65	12.2	-	32.1	40.3	84.7	-27	-2	-1	8.3	17.5	49.8	-33	+ 37	+ 20	69.7	+ 3
66–75	6.9	-	36.3	49.2	89.8	-30	0	+ 1	14.5	27.6	64.2	-26	+ 14	+ 13	97.0	+ 11
76–85	4.2	-	43.6	54.5	90.0	-28	-1	0	23.8	35.2	71.1	-20	+ 3	+ 6	170.4	+ 9
> 85	1.8	-	41.6	46.1	77.4	-23	+ 2	+ 3	29.2	33.5	61.2	-7	+ 8	+ 10	224.7	+ 11
**Sex**																
Male	30.7	40.6	23.3	29.7	77.1	-33	-8	-3	6.5	13.6	37.2	-26	+ 41	+ 34	296.1	+ 10
Female	33.5	43.1	28.4	37.1	84.0	-34	-6	-2	8.2	16.7	45.3	-29	+ 37	+ 25	290.9	+ 8
**Geographic deprivation index (quintile)**																
Q1 (Most favored)	12.6	41.2	22.0	29.7	77.9	-38	-9	-4	5.8	12.9	39.0	-30	+ 45	+ 38	95.0	+ 13
Q2	12.9	41.2	25.6	33.3	81.4	-34	-7	-2	6.9	14.7	41.1	-28	+ 44	+ 32	102.0	+ 9
Q3	12.9	42.1	26.5	34.1	81.4	-33	-6	-2	7.7	15.4	41.6	-28	+ 38	+ 28	118.0	+ 8
Q4	12.6	42.8	27.5	34.9	81.7	-32	-6	-3	8.3	16.3	42.7	-27	+ 35	+ 24	128.1	+ 8
Q5 (Least favored)	12.2	42.3	28.6	36.5	81.8	-32	-6	-3	8.6	16.9	43.4	-28	+ 33	+ 23	135.0	+ 9
**Covid-19 hospitalization rate, LD 1**																
Q1^a^ < 54.8	11.3	43.6	27.5	34.5	82.3	-32	-7	-2	8.5	16.3	43.3	-25	+ 32	+ 25	109.5	+ 3
Q2 54.8–88.1	15.4	42.7	26.5	34.2	81.9	-33	-6	-2	7.5	15.1	40.6	-25	+ 39	+ 31	143.9	+ 6
Q3 88,1–178.1	13.3	42.4	27.6	35.5	82.0	-33	-6	-2	8.3	17.3	44.6	-29	+ 41	+ 27	132.5	+ 9
Q4 > 178.1	24.0	40.2	24.0	31.7	78.5	-35	-7	-3	6.4	13.5	39.4	-31	+ 41	+ 30	200.6	+ 15
**Covid-19 hospitalization rate, 2020**																
Q1^a^ < 233.2	12.9	43.3	27.3	34.3	82.4	-32	-7	-2	8.3	16.2	43.5	-25	+ 32	+ 26	121.2	+ 3
Q2 233.2–352.8	13.6	43.3	27.0	34.8	81.9	-33	-7	-2	7.7	15.1	39.8	-25	+ 34	+ 27	130.8	+ 5
Q3 352.8–511.0	17.8	41.1	26.1	33.9	80.7	-33	-7	-3	7.4	15.7	42.4	-30	+ 43	+ 29	160.9	+ 12
Q4 > 511.0	19.9	40.8	24.2	32.0	78.8	-35	-7	-3	6.7	14.1	40.5	-31	+ 43	+ 32	173.7	+ 15

LD 1: First national lockdown W11-W18 (March 17 to May 10, 2020)

LD 2: Second national lockdown W44-W49 (October 30 to December 14, 2020)

^a^Quartile by are of residence, rate of hospitalization for COVID-19 per 100,000 people

**Table 3:** Description of person characteristics with at least one consultation with physiotherapists and dentists during the lockdown periods and the whole of 2020 relative to the corresponding periods in 2019, for mainland France

		**Physiotherapist**						**Dentist**					
		**At least one consultation 2020**			**Ratio 2020/2019**			**At least one consultation 2020**			**Ratio 2020/2019**		
	**Individuals**	LD 1	LD 2	Year	LD 1	LD 2	Year	LD 1	LD 2	Year	LD 1	LD 2	Year
	2020 Million	%	%	%	%	%	%	%	%	%	%	%	%
**Total**	64.3	1.6	6.7	15.2	-80	-3	-8	0.5	9.8	37.1	-95	1	-12
**Age (years)**													
0–1	1.4	0.6	1.1	7.0	-86	-44	-44	0.0	0.5	1.7	-95	-3	-14
2–6	3.7	0.1	0.5	1.2	-83	-11	-24	0.2	7.1	29.9	-97	-5	-13
7–17	8.4	0.3	2.4	6.2	-89	-9	-17	0.9	9.2	40.1	-92	0	-15
18–25	5.8	0.5	3.0	8.5	-86	+ 2	-7	0.4	6.9	30.5	-95	+ 8	-12
26–50	20.0	1.1	6.1	14.5	-84	0	-9	0.6	10.6	39.1	-95	+ 4	-13
51–65	12.2	2.0	9.1	20.0	-81	-2	-7	0.6	12.3	42.8	-96	0	-11
66–75	6.9	2.5	10.2	22.7	-79	-5	-6	0.5	11.6	41.3	-96	0	-9
76–85	4.2	4.5	13.5	27.9	-72	-7	-7	0.4	8.8	33.8	-96	-5	-11
> 85	1.8	8.6	16.6	31.0	-57	-2	-1	0.2	4.0	18.0	-97	-8	-12
**Sex**													
Male	30.7	1.3	5.1	12.5	-79	-3	-9	0.5	9.0	34.4	-95	+ 1	-12
Female	33.5	1.9	8.1	17.7	-80	-3	-8	0.5	10.5	39.6	-95	+ 1	-12
**Geographic deprivation index (quintile)**													
Q1(Most favored)	12.6	1.7	7.4	16.3	-80	-3	-7	0.5	10.6	40.7	-95	+ 3	-11
Q2	12.9	1.8	7.4	16.6	-80	-2	-8	0.5	10.3	38.8	-95	+ 2	-12
Q3	12.9	1.7	7.1	16.1	-79	-2	-8	0.5	9.9	37.5	-95	+ 1	-12
Q4	12.6	1.5	6.2	14.4	-79	-4	-9	0.6	9.4	35.4	-95	-1	-13
Q5 (Least favored)	12.2	1.4	5.4	12.6	-79	-5	-10	0.5	8.8	33.1	-95	-2	-14
**Covid-19 hospitalization rate, LD 1**													
Q1^a^ < 54.8	11.3	1.8	7.2	16.5	-79	-2	-8	0.5	10.0	37.6	-95	0	-13
Q2 54.8–88.1	15.4	1.9	7.5	16.9	-78	-2	-8	0.5	9.8	37.4	-95	+ 1	-12
Q3 88.1–178.1	13.3	1.8	6.7	15.1	-78	-4	-9	0.6	9.5	35.7	-95	-1	-14
Q4 > 178.1	24.0	1.3	5.9	13.5	-82	-4	-8	0.5	9.9	37.5	-95	+ 3	-11
**Covid-19 hospitalization rate, 2020**													
Q1^a^ < 233.2	12.9	1.8	7.3	16.5	-79	-1	-8	0.5	10.0	37.7	-96	+ 1	-13
Q2 233.2–352.8	13.6	1.9	7.3	16.5	-78	-3	-8	0.6	9.8	36.9	-95	+ 1	-13
Q3 352.8–511.0	17.8	1.6	6.2	14.0	-78	-5	-9	1.6	9.4	35.7	-95	0	-13
Q4 511.0 and above	19.9	1.3	6.3	14.5	-82	-3	-8	1.3	10.1	38.2	-95	+ 2	-11

LD 1: First national lockdown W11-W18 (March 17 to May 10, 2020)

LD 2: Second national lockdown W44-W49 (October 30 to December 14, 2020)

^a^Quartile by area of residence, rate of hospitalization for COVID-19 per 100,000 people

For the entire year of 2020, the decreases in the consultations were greatest for the youngest individuals: physiotherapists (0–1 year: -44%, 2–6 years: -24%, 7–17: years -17%), GPs ( 0–1 year: -7%, 2–6 years: -9%, 7–17 years: -6%). For dentists, the decline was consistent across age groups. Conversely, children displayed the largest increase in nurse consultations (+ 102% for 7–17-year-olds), despite being much less likely to use nursing care than older people. Among adults, the decrease in GP and physiotherapist consultations decreased with age, disappearing altogether for elderly patients, as observed for nurse consultations. The change in the proportion of people with at least one consultation with a specialist (Table 4) between 2019 and 2020 was essentially negative for all age groups, except for psychiatrist consultations for 18- to 25-year-olds. For the youngest individuals, the deficits were highest for ENT specialists, pulmonologists, dermatologists, and ophthalmologists. The oldest individuals were less likely to have had at least one consultation with a rheumatologist, gastroenterologist, ENT specialist, or cardiologist than in 2019. The decreases were similar according to sex, social-deprivation quintile, and COVID-19 hospitalization quartile.

**Table 4:** Description of person characteristics with at least one referral to medical specialists in 2020 relative to 2019 in mainland France

	**Individual**	**Ophthalmologist**		**Gynecologist**		**Dermatologist**		**Cardiologist**		**ORL**	
	**2020**	**Consul**^a^	**2020/2019**^b^	**Consul**	**2020/2019**	**Consul**	**2020/2019**	**Consul**	**2020/2019**	**Consul**	**2020/2019**
	Million	%	%	%	%	%	%	%	%	%	%
**Total**	64.3	26.7	-7	10.2	-9	9.1	-12	9.6	-5	6.3	-11
**Age (years)**											
0–1	1.4	6.2	-13	0.1	31	2.4	-17	0.7	-7	5.4	-26
2–6	3.7	21.8	-10	0.0	+ 3	3.5	-18	0.7	-17	8.9	-24
7–17	8.4	26.7	-7	2.0	-9	7.5	-13	1.3	-15	4.0	-15
18–25	5.8	17.4	-7	13.1	-7	7.8	-6	1.9	-2	3.7	-4
26–50	20.0	22.1	-9	17.9	-9	8.6	-12	4.5	-6	5.2	-8
51–65	12.2	31.3	-7	11.5	-10	10.1	-13	13.8	-6	7.3	-10
66–75	6.9	39.9	-4	7.3	-12	13.5	-10	25.0	-3	9.3	-7
76–85	4.2	39.8	-7	3.2	-11	12.7	-11	30.8	-5	10.1	-10
> 85	1.8	22.9	-8	0.7	-9	7.0	-12	20.2	-5	6.9	-11
**Sex**											
Male	30.7	23.3	-7	0.2	+ 6	7.5	-11	10.4	-5	6.2	-10
Female	33.5	29.8	-8	19.4	-9	10.3	-12	8.9	-5	6.5	-11
**Geographic deprivation index (quintile)**											
Q1 (Post favored)	12.6	28.8	-7	13.6	-9	12.5	-11	10.1	-5	7.7	-11
Q2	12.9	27.3	-7	11.1	-9	9.8	-11	9.6	-5	6.5	-10
Q3	12.9	26.7	-8	10.1	-9	8.9	-11	9.8	-5	6.3	-10
Q4	12.6	26.1	-7	8.6	-10	7.4	-13	9.3	-5	5.7	-11
Q5 (Least favored)	12.2	25.0	-7	7.6	-11	6.3	-14	9.4	-6	5.6	-12
**Covid-19 hospitalization rate, 2020**											
Q1^c^ < 233.2	12.9	27.3	-8	8.7	-10	8.2	-12	9.2	-5	5.7	-11
Q2 233.2–352.8	13.6	27.1	-8	8.7	-10	8.2	-12	9.2	-5	5.7	-11
Q3 352.8–511.0	17.8	25.9	-8	9.5	-10	9.3	-12	9.8	-6	6.2	-11
Q4 > 511.0	19.9	26.8	-7	10.3	-9	8.1	-12	9.3	-5	6.1	-11

	**Gastroenterologist**		**Rheumatologist**		**Pediatrician**		**Pulmonologist**		**Psychiatrist**		**Endocrinologist**	
	**Consul**	**2020/2019**	**Consul**	**2020/2019**	**Consul**	**2020/2019**	**Consul**	**2020/2019**	**Consul**	**2020/2019**	**Consul**	**2020/2019**
	%	%	%	%	%	%	%	%	%	%	%	%
**Total**	3.9	-7	3.2	-10	3.6	-4	2.8	-4	2.4	-3	1.6	-4
**Age (years)**												
0–1	0.0	-37	0.0	+ 2	45.6	-4	0.1	-45	0.1	-6	0.0	-15
2–6	0.1	-32	0.0	-12	24.3	-6	0.7	-30	0.7	-15	0.0	-9
7–17	0.4	-16	0.2	-16	7.3	-2	1.2	-12	1.3	-7	0.2	-9
18–25	1.9	-4	0.6	-6	0.5	-2	1.1	1	2.0	+ 3	1.0	-2
26–50	3.8	-7	2.3	-9			2.0	1	3.3	-5	1.8	-3
51–65	6.5	-8	5.5	-10			4.2	-3	3.5	-2	2.5	-4
66–75	8.0	-6	7.4	-10			5.8	-2	1.8	-1	3.0	-3
76–85	5.8	-9	7.6	-12			5.5	-6	1.2	-3	2.2	-4
> 85	2.2	-8	3.6	-10			2.5	-6	0.7	-4	0.8	-3
**Sex**												
Male	3.7	-7	2.2	-10	3.7	-4	3.0	-4	1.9	-4	1.0	-2
Female	4.2	-8	4.2	-10	3.5	-4	2.5	-3	2.8	-3	2.3	-4
**Geographic deprivation index (quintile)**												
Q1 (Post favored)	4.5	-7	3.8	-9	5.2	-3	2.6	-3	3.3	-5	2.1	-5
Q2	4.1	-7	3.4	-10	3.8	-4	2.8	-3	2.5	-3	1.8	-3
Q3	4.0	-7	3.3	-10	3.5	-4	2.9	-4	2.5	-3	1.7	-3
Q4	3.6	-8	3.0	-11	2.8	-5	2.8	-4	1.9	-3	1.3	-4
Q5 (Least favored)	3.4	-8	2.8	-12	2.8	-6	2.7	-4	1.6	-3	1.3	-3
**Covid-19 hospitalization rate, 2020**												
Q1^c^ < 233.2	4.0	-7	3.0	-9	2.7	-3	2.7	-4	2.1	-3	1.4	-3
Q2 233.2–352.8	4.0	-7	3.0	-9	2.7	-3	2.7	-4	2.1	-3	1.4	-3
Q3 352.8–511.0	3.9	-7	3.5	-9	3.2	-3	3.0	-5	2.5	-5	1.8	-5
Q4 > 511.0	3.9	-8	2.8	-12	3.4	-5	2.9	-3	2.0	-2	1.4	-3

Consul: percentage of patients with at less one consultation in 2020

2020/2019: difference between the two years in the percentage of patients with at least one annual consultation

^a^Proportion of persons with at least one consultation in 2020

^b^Ratio

^c^Quartile by area of residence, rate of hospitalization for COVID-19 per 100,000 people

### Consultations according to the medical condition of the individual

For medical conditions identified in 2019, 42% of individuals (mean age 60.4 years) had at least one consultation decrease with a GP during the first lockdown, a decrease of 24% relative to 2019. For the entire year of 2020, the corresponding values were 89% and + 1%, respectively (Table 5). For nurses, the corresponding values were 19% and -18% for the first confinement and 62% and + 14% for the entire year of 2020 (i.e., more individuals with at least one consultation than in 2019). Depending on the medical condition identified, the percentage of patients with at least one consultation with a GP during the first confinement also decreased, but to a lesser extent, particularly for cardiovascular diseases (46%, -22%), cancer (41%, -24%), psychiatric illnesses (42%, -22%), and neurological or degenerative diseases (39%, -23%). Over the entire, the proportion of patients with at least one GP consultation was higher, and close to that of 2019, but rarely exceeded 90%. The decrease in the number of nurse consultations during the first lockdown was smaller than the decrease in GP consultations. There were generally more consultations than in 2019 for the entire year and for all medical conditions, particularly for individuals with at least one psychiatric illness, whose mean age was lower than that of the total population. The decrease in GP consultations during the first lockdown was smaller for individuals with a high MRMI (reflecting a higher risk of mortality) than for those with the lowest levels of comorbidity (low index values). The opposite was true for nurses, for the year as a whole.

**Table 5:** Description of consultations with GPs and nurses during the first lockdown and for the whole of 2020 relative to the corresponding periods in 2019 and deaths in 2020 in mainland France, as a function of the disease identified in the 2019 and a morbidity index

			**General practitioner**				**Nurse**				**Deaths**	
			**At least one consultation 2020**		**Ratio 2020/2019**		**At least one consultation 2020**		**Ratio 2020/2019**		**2020**	**Ratio 2020/2019**
	**Individuals**	**Age**	**LD 1**	**2020**	**LD 1**	**2020**	**LD 1**	**2020**	**LD 1**	**2020**		
	2020%	mea*n*	%	%	%	%	%	%	%	%	/100.000	%
**Total**	**100.0**	**41.9**	**26.0**	**80.7**	**-34**	**-3**	**7.4**	**41.5**	**-28**	** + 29**	**0.9**	** + 9**
**No medical condition**	**75.0**	**35.7**	**20.7**	**78.1**	**-39**	**-4**	**3.4**	**34.6**	**-42**	** + 40**	**0.1**	** + 6**
**At least one medical condition**	**25.0**	**60.4**	**41.7**	**88.6**	**-24**	** + 1**	**19.4**	**62.1**	**-18**	** + 14**	**3.2**	** + 9**
**Heart disease**	**7.8**	**72.4**	**46.2**	**87.5**	**-22**	** + 2**	**26.0**	**67.9**	**-14**	** + 11**	**5.8**	** + 9**
Coronary disease	3.2	72.3	47.6	89.5	-21	+ 2	24.4	69.8	-15	+ 11	5.1	+ 9
Stroke	1.4	72.2	44.1	83.1	-22	+ 2	25.6	62.8	-11	+ 12	6.9	+ 11
Heart failure	1.3	79.3	43.6	74.8	-20	0	34.2	62.6	-8	+ 6	12.3	+ 5
Lower limb arteriopathy	1.1	73.3	47.9	86.6	-22	0	28.0	68.9	-14	+ 7	7.6	+ 8
Rhythm or conduction disturbances	2.7	76.2	46.0	83.8	-21	+ 2	31.0	68.3	-12	+ 9	7.8	+ 7
Valvular disease	0.7	75.7	47.1	84.6	-21	+ 1	34.6	70.8	-10	+ 7	8.1	+ 6
Acute pulmonary embolism	0.1	69.6	43.8	78.4	-20	0	28.1	63.0	-12	+ 9	8.3	+ 5
**Cancers**	**5.1**	**69.1**	**40.7**	**85.7**	**-24**	** + 1**	**25.4**	**69.1**	**-13**	** + 11**	**5.7**	** + 6**
Actively treated	2.2	68.4	39.9	80.8	-22	0	31.7	69.8	-9	+ 8	8.5	+ 2
Monitored	3.0	69.9	41.4	89.1	-24	+ 2	21.1	68.6	-18	+ 13	3.8	+ 13
**Psychiatric illnesses**	**3.8**	**53.5**	**42.2**	**84.7**	**-22**	**0**	**18.6**	**54.1**	**-14**	** + 15**	**3.4**	** + 11**
Psychotic disorders	0.7	51.4	36.5	78.7	-23	0	23.4	52.6	-6	+ 13	2.9	+ 14
Neurotic and mood disorders	2.2	58.3	47.6	88.5	-20	+ 1	20.6	59.8	-15	+ 14	3.6	+ 11
Manic and bipolar disorders	0.4	56.1	44.6	88.6	-20	+ 2	20.6	61.2	-14	+ 15	2.3	+ 14
Depression and mood disorders	1.4	58.9	49.4	89.6	-21	-1	20.5	60.6	-17	+ 12	3.5	+ 8
Neurotic disorders	0.8	57.8	47.3	87.2	-19	+ 2	22.8	59.9	-9	+ 16	4.7	+ 15
Mental impairment	0.2	41.4	31.6	77.0	-29	-2	14.2	44.3	-15	+ 22	2.0	+ 17
Addictive disorders	0.7	51.0	42.9	82.4	-17	0	17.6	52.5	-13	+ 14	3.8	+ 6
Childhood-onset disorders	0.3	17.2	21.8	80.2	-38	+ 3	4.6	28.0	-20	+ 56	0.4	+ 12
**Neurological or degenerative diseases**	**2.5**	**69.2**	**39.1**	**77.3**	**-23**	**0**	**22.9**	**53.9**	**-9**	** + 13**	**9.6**	** + 13**
Dementia	1.2	84.9	37.0	66.4	-24	-2	25.9	47.1	-4	+ 11	16.2	+ 12
Parkinson’s disease	0.4	76.9	45.5	83.3	-23	0	27.5	64.0	-9	+ 10	9.8	+ 13
Multiple sclerosis	0.2	51.9	41.7	89.8	-20	+ 2	21.4	68.4	-19	+ 15	1.3	+ 2
Paraplegia	0.1	53.9	40.9	82.9	-21	+ 1	26.4	61.7	-10	+ 12	4.1	+ 5
Myopathy or myasthenia gravis	0.1	51.9	39.1	87.7	-24	+ 1	18.7	61.5	-19	+ 16	2.8	+ 5
Epilepsy	0.5	49.2	37.8	83.7	-24	+ 1	16.5	51.0	-15	+ 17	4.1	+ 10
**Chronic respiratory diseases**	**5.5**	**52.5**	**42.7**	**90.1**	**-25**	**-1**	**15.7**	**55.5**	**-20**	** + 16**	**3.1**	** + 6**
**Cystic fibrosis**	**0.0**	**27.6**	**21.9**	**74.1**	**-34**	**-3**	**17.6**	**58.4**	**-13**	** + 17**	**1.4**	** + 5**
**HIV**	**2.0**	**56.4**	**41.6**	**88.7**	**-19**	** + 3**	**22.9**	**67.5**	**-15**	** + 17**	**2.1**	** + 12**
**Diseases of the liver or pancreas**	**0.9**	**59.9**	**40.9**	**82.1**	**-20**	**0**	**22.2**	**29.0**	**-14**	** + 7**	**5.0**	** + 7**
**Rheumatoid arthritis, related diseases**	**0.5**	**65.1**	**45.7**	**90.8**	**-21**	** + 2**	**31.1**	**75.7**	**-15**	** + 11**	**3.0**	** + 10**
**Hemophilia, hemostasis disorders**	**0.1**	**53.3**	**42.4**	**90.4**	**-20**	** + 4**	**27.8**	**67.4**	**-10**	** + 17**	**2.0**	** + 16**
**Diabetes**	**5.9**	**67.6**	**48.2**	**92.3**	**-21**	** + 2**	**27.3**	**38.4**	**-16**	** + 11**	**3.6**	** + 14**
**Chronic dialysis**	**0.1**	**70.6**	**25.6**	**67.6**	**-29**	**-1**	**30.5**	**56.5**	**-2**	** + 8**	**13.5**	** + 7**
**Kidney transplant before 2020**	**0.1**	**56.6**	**31.6**	**81.3**	**-20**	**0**	**42.9**	**79.7**	**18**	** + 16**	**3.9**	** + 23**
**Mortality-Related Morbidity Index**												
0	2.1	67.0	29.5	88.2	-39	-3	6.7	19.8	-47	+ 12	0.2	+ 8
1	4.0	69.1	32.4	88.9	-34	+ 1	9.2	22.9	-37	+ 15	0.4	+ 9
2	3.7	72.1	37.6	91.7	-30	+ 2	13.4	27.8	-29	+ 13	0.8	+ 16
3	3.1	75.7	42.0	93.0	-29	+ 1	18.3	32.4	-25	+ 10	1.5	+ 10
4	2.6	79.2	44.6	92.2	-27	+ 1	24.1	36.8	-20	+ 7	3.0	+ 12
5	2.0	82.1	46.3	89.5	-25	+ 1	30.4	40.3	-14	+ 7	5.5	+ 10
6	1.5	84.4	46.1	84.2	-22	+ 2	34.7	40.5	-8	+ 8	9.3	+ 11
7	1.0	86.2	44.0	77.2	-21	+ 2	34.9	37.1	-5	+ 9	13.3	+ 11
8	0.6	87.5	40.7	70.0	-20	+ 1	32.9	32.6	-3	+ 9	16.4	+ 10
9	0.6	88.8	36.1	60.8	-19	0	30.0	27.4	-2	+ 9	19.7	+ 8

LD 1: First national lockdown W11-W18 (March 17 to May 10, 2020)

### Deaths according to patient characteristics and diseases

There were 9% more deaths in 2020 than in 2019: + 3% for individuals between 51 and 65 years of age and + 10% for older individuals (Tables 2 and 3). The increase in the number of deaths was larger in the more advantaged regions (i.e., + 13% for the first quintile of the social deprivation index versus + 8% and + 9% for the other four, less advantaged quintiles), despite similar mean ages. The excess was + 15% for the quartile of departments with the highest rates of hospitalization for COVID-19, versus + 3% for those with the lowest rates of COVID-19 and a slightly older population. There were 9% excess deaths among people with at least one medical condition among those studied (Table 5): + 9% for cardiovascular disease and + 6% for cancer (cancer in the active phase of treatment: + 2%, monitored: + 13%). For individuals with psychiatric illnesses, the excess death rate was 11%. It was particularly high for those with psychotic (+ 14%, mean age 51 years), manic and bipolar (+ 14%, 56 years), and neurotic (+ 15%, 58 years) disorders and mental impairment (+ 17%, 41 years). The excess death rate was + 12% for dementia (patients with a mean age of 85 years). For the MRMI, mortality in 2020 increased with the value of the index, but the number of excess deaths rose and then remained relatively stable, at about + 10%.

## Discussion

This French national observational cohort study on an almost exhaustive population highlights a decrease in the use of healthcare and in the number of consultations, to various extents according to the primary care specialty, and changes in the levels of certain activities, such as screening, relative to 2019, especially during the first lockdown in the spring of 2020 and, to a lesser extent, for the year overall. A particularly large decrease was observed for two- to 17-year-olds. The amplitude of the decrease diminished with age and the presence of medical conditions, but without a return to the annual figures of 2019 in some cases. Teleconsultations were almost non-existent in 2019, but took off strongly during the first confinement, limiting the decrease in consultations, but to different extents for different specialties. The death rate was generally higher in 2020 than in 2019 (9%), especially among the elderly and for individuals with certain medical conditions.

The number of consultations with professionals, which had remained stable since the beginning of the year, might have been expected to increase following the announcement of the first lockdown, but the opposite was observed, with a decrease right from the start of lockdown. This can be explained by the short period of five days between lockdown being announced and coming into force and the fears of the population in the face of the rapidly growing first wave of the epidemic [13, 14]. However, a study on the same population reported a strong growth (+ 20% to + 40%) in the delivery of certain classes of drugs at the start of the first lockdown [28]. The drugs concerned were particularly those indicated for chronic diseases, and the intention may have been to stockpile them. In France, such drugs can be delivered without consultation if there is already a medical prescription and depending on the duration of the prescription. This may partially explain the absence of a peak in consultations, at least for the obtainment or renewal of prescriptions. In addition, the duration of validity of prescriptions was extended and their renewal was also facilitated by the sharp increase in teleconsultations. Nevertheless, the same study reported also a lower rate of initiation for cardiovascular and anti-diabetic treatments during this period [29].

Post-lockdown, once drug delivery rates had returned to normal, no such difference was observed during the second confinement. For consultations, no rebound phenomenon such as might have been expected after 55 days of lockdown was observed, but the context remained restrictive, and the release from lockdown also corresponded to a period containing a large number of public holidays. The time between the announcement of the second lockdown and its coming into effect was even shorter (2 days) than for the first lockdown, but the implementation of this second lockdown was preceded by a peak in the number of consultations. This may reflect an anticipation of the lockdown, favored by the gradual extension of curfews and a slower progression of the second wave in a population now familiar with this context. During the second lockdown, which was less strict than the first, with, in particular, a greater availability of protective equipment and screening tests, the decrease in the use of primary healthcare was less marked.

By the end of 2020, healthcare use was clearly lower than that in 2019, whether few considered single consultations or the total number of consultations, for almost all professions. This was especially true for dentists and physiotherapists, and for ENT specialists, dermatologists, gastroenterologists, ophthalmologists, rheumatologists, and general practitioners. The usual modes of examination and care, requiring close contact with the patient, simultaneous or group treatment, and specific equipment, may have influenced the number of consultations due to the lack of availability of specific protection and protocols. It was the youngest, in particular, who experienced the largest annual decrease in GP consultations, as in Canada [17], and the largest decreases were observed for dentists, physiotherapists, and certain medical specialists. However, for certain medical specialties, there were fewer young patients, such as those with seasonal infections or sports-related or accidental injuries, especially during the periods of school closure but also scheduled follow-ups or prevention visits and those not really necessary [30] The impact of the decrease in dentistry consultations should be investigated further, as should the potential decrease in consultations for compulsory examinations and vaccinations [18–20] or for chronic disease follow-up, as in adults [21]. The lower rates of consultation for the youngest patients may have favored a transfer of the activities of healthcare professionals to the oldest individuals or the elderly people with chronic diseases, thus limiting the decrease in consultation in this age group, as observed in Canada [21, 24]. In addition, the decreases in consultation rates were substantial for certain conditions, but it was possible to carry out consultations in specialist centers, for renal replacement treatment in patients with kidney failure and for specialist treatments for cystic fibrosis, for example. This may also have been the case for the very elderly in nursing homes or for those who institutionalized, in particular, for nursing care paid on a flat-rate basis.

The increasing availability and spread of screening tests may account for the increase in the number of nurse consultations in 2020, beginning in the summer and peaking before the second confinement, and, to a lesser extent, for midwives. Indeed, decrees published at the end of July 2020 authorized practitioners of these professions to take samples from patients suspected of having COVID-19 without a medical prescription, and then, in the second half of October, to perform COVID-19 antigenic tests. This last point may account for the high level of activity at the end of the year, with many people choosing to be screened before family gatherings. Conversely, the lower use of GPs and pediatricians relative to 2019 may be explained by a number of factors, including the adoption of barrier measures, which probably limited the spread of winter infectious diseases [31].

In many countries, GPs modified and adapted their practices by developing teleconsultation during the first half of 2020 [18–21]. In France, this practice was authorized and implemented in June 2018, but it was little used. Full reimbursement (100%) for teleconsultation was introduced in March 2020 and has been extended to 2022, and the possibility of a telephone-only consultation was authorized from April to May 2020. The specialties with the smallest decreases in the number of consultations were those with the largest proportions of teleconsultations, such as GPs, endocrinologists, and, especially, psychiatrists. The use of teleconsultations declined after the first lockdown, with a slight rebound during the second lockdown. Teleconsultation was well accepted, bue does not allow the possibility of a classic clinical examination, with the risk of underdiagnosis and low rates of treatment initiation, for diabetes or arterial hypertension, for example [17, 19]. The preservation of relatively high levels of activity for psychiatrists may have been favored by the greater use teleconsultation, and by the decompensation of known psychological disorders or their development in the population [32].

The reasons for consultations are not recorded in France, but changes in the reasons for consultation during the pandemic have been reported in other countries, depending on the type of consultation (remote or in person) in particular [33–35]. In the USA, consultations for classic infectious diseases decreased for the first six months of the pandemic, whereas, for teleconsultation, the reasons for consultation were mostly non-specific infections or the monitoring of mental disorders [16]. In Germany, the number of consultations concerning the digestive system, dizziness, and spinal disorders, fatigue and general weakness decreased for adults [19]. In Canada, the number of visits for diabetes and hypertension was affected, as was the number of visits relating to prevention, and teleconsultations focused principally anxiety and depressive disorders [17]. These studies also suggested a decrease in the number of consultations linked to undefined symptoms and, therefore, probably lacking a real motivation. In addition, the frequency of numerous follow-up biological examinations, endoscopies, and specialized surgical interventions decreased, as did the quality-of-care indicators relating to the follow-up of chronic diseases, possibly linked to the smaller number of consultations [16, 19, 20, 36].

Leaving aside their role in in COVID-19 screening activity later in the year, nursing was the profession for which consultations decreased the least during the first lockdown, with an increase in the mean number of consultations per patient, particularly for older patients. This may be linked to the need to maintain care continuity, and an increase in the need for care for fragile patients and patients discharged earlier from hospital due to the epidemic context [37, 38].

The underuse of midwives early in the year was reversed by the end of the year, possibly due to the combined effects of a role in screening activities later in the year and less intense monitoring of pregnant women and of children born at the start of 2020. A qualitative study of midwives in France found evidence of a postponement or cancellation of non-essential care, such as postpartum perineal rehabilitation, preparation of the birth, preventive gynecological care, prenatal interview and postnatal follow-up [37, 39]. A lower rate of preterm deliveries and stillbirths was also reported after the onset of the epidemic, consistent with a decrease in the factors favoring preterm birth [39, 40].

There were fewer consultations with physiotherapists than in 2019, but the mean number of consultations per person did not decrease significantly. This may reflect an optimization, with a redirection of activities towards individual care of the elderly or severely disabled, facilitated by practice within institutions, and a decrease in activities associated with a risk of injury to the musculoskeletal system [40, 41]. Nevertheless, there may have been a demand for specific post-hospital rehabilitative care for COVID-19 patients [41, 42].

There was a slight decrease in the number of GP and nurse consultations in areas for which the intensity of the epidemic was high during the first lockdown, as reported elsewhere [17]. However, no further deficit was observed for nurses after the first wave, consistent with more intensive care and COVID-19 screening activities due to the high intensity of the epidemic. Residents of the least socially disadvantaged geographic areas had fewer consultations with nurses and GPs than those in the most disadvantaged areas during the first lockdown. However, for the year as a whole, the more socially disadvantaged areas presented the greatest excess use of nurses, possibly linked to more intense COVID-19 screening in these areas.

In France, 56,000 excess deaths from all causes and for all places of death were recorded by the INSEE in 2020 (i.e., 9% more than in 2019), as in this study, mostly in individuals over the age of 65 years [23]. For non-exclusive conditions identified in 2019 and mostly affecting relatively elderly individuals, excess death rates were mostly about 9%. However, they were higher for chronic conditions identified as potential risk factors for death due to COVID-19: monitored cancer (without active treatment), cystic fibrosis, HIV infection, hemostasis disorders, diabetes, hemophilia, and certain psychiatric illnesses, such as mental impairment [9]. People with psychiatric illnesses had higher excess death rates at a relatively young age. This can be attributed to a worsening or decompensation of their illness, a greater risk of being infected or of developing more severe COVID-19, or the presence of comorbidities, of a cardiovascular nature in particular, which are more frequent in this population, with poorer access to somatic care even in the absence of the pandemic [42, 43]. For psychiatric illnesses, the decrease in the use of primary care was not greater than for other conditions, except for mental impairment and childhood-onset disorders, particularly during the first lockdown. It is possible that these people are institutionalized in care homes with specific integrated medical care. Further studies are required to characterize the link between the under-use of care and the occurrence of complications and death in more detail.

### Limitations

This study concerns a large, almost exhaustive population, but one treated in the context of a specific healthcare system that provides near-universal health coverage, which may limit extrapolation to other countries or healthcare systems, although many are similar. The amount and density of primary care facilities available may affect their level of use. However, these factors were similar in 2019 and 2020. We only include out-of-hospital visits and not hospital outpatient visits in our analysis. Such consultations are less numerous than those with private practitioners, but may also have decreased sharply in 2020, given the health context and the changes in care organization. Moreover, lessons and adaptations have to be drawn from these different analyses and modulated according to the specificities of the different health systems.

## Conclusions

The decrease in consultations with primary care professionals in 2020 was substantial, especially during the first lockdown, despite strong teleconsultation activity, with differences according to age and medical specialty. The short- and long-term consequences of this decrease in consultations should be investigated, in terms of morbidity and mortality, and the analysis of fluctuations should be continued for 2021 in light of the appearance of new epidemic waves.

## Data Availability

The data are available on request from the corresponding author upon receipt of a statistical analysis plan addressing an important new scientific question, approved by the COPE Study Steering Committee.

## Abbreviations

ALD: Long term chronic disease (*affection de longue durée*)
Covid-19: Coronavirus disease 19
DROM: French overseas territories (départements et régions d’outremer)
ENT: Ear, nose, and throat
GP: General practitioner
HIV: Human immunodeficiency virus
ICD-10: International Classification of Diseases 10^th^ revision
INSEE: National Institute for Statistics and Economic Studies (*Institut National de la Statistique et des Études Économiques*-)
LD: Lockdown
MRMI: Mortality-Related Morbidity Index
SNDS: National health database (*système national des données de santé*)

## References

[1] Flaxman S, Mishra S, Gandy A, Unwin JT, Mellan TA, Coupland H (2020). Estimating the effects of non-pharmaceutical interventions on COVID-19 in Europe. Nature.

[2] Jeffery M, D'Onofrio G, Paek H, Platts-Mills TF, Soares WE, Hoppe JA (2020). Trends in emergency department visits and hospital admissions in health care systems in 5 states in the first months of the COVID-19 pandemic in the US. JAMA Intern Med.

[3] Bollmann A, Hohenstein S, Pellissier V, Stengler K, Reichardt P, Ritz JP (2021). Utilization of in- and outpatient hospital care in Germany during the COVID-19 pandemic insights from the German-wide Helios hospital network. PLoS ONE.

[4] Santi L, Golinelli D, Tampieri A, Farina G, Greco M, Rosa S (2021). Non-COVID-19 patients in times of pandemic: Emergency department visits, hospitalizations and cause-specific mortality in Northern Italy. PLoS ONE.

[5] Gabet A, Grave C, Tuppin P, Chatignoux E, Béjot Y, Olié V (2021). Impact of the COVID-19 pandemic and a national lockdown on hospitalizations for stroke and related 30-day mortality in France: A nationwide observational study. Eur J Neurol.

[6] Gabet A, Grave C, Tuppin P, Emmerich J, Olié V (2021). Changes in the epidemiology of patients hospitalized in France with venous thrombosis and pulmonary embolism during the COVID-19 pandemic. Thromb Res.

[7] Grave C, Gabet A, Puymirat E, Empana JP, Tuppin P, Danchin N (2021). Myocardial infarction throughout one year of COVID-19 pandemic: French nationwide study of hospitalisation rates, prognostic and 90-day mortality rates. Arch Cardiovasc Dis.

[8] Griffin S (2020). Covid-19: “Staggering number” of extra deaths in community is not explained by COVID-19. BMJ.

[9] Semenzato L, Botton J, Drouin J, Cuenot F, Dray-Spira R, Weill A (2021). Chronic diseases, health conditions and risk of COVID-19-related hospitalization and in-hospital mortality during the first wave of the epidemic in France: a cohort study of 66 million people. Lancet Reg Health Eur.

[10] Karmakar M, Lantz PM, Tipirneni R (2021). Association of social and demographic factors with COVID-19 incidence and death rates in the US. JAMA Netw Open.

[11] Brugel M, Carlier C, Essner E, Debreuve-Theresette A, Beck MF, Merrouche Y (2021). Dramatic changes in oncology care pathways during the COVID-19 pandemic: The French ONCOCARE-COV Study. Oncologist.

[12] Søreide K, Hallet J, Matthews JB, Schnitzbauer AA, Line PD, Lai PBS (2020). Immediate and long-term impact of the COVID-19 pandemic on delivery of surgical services. Br J Surg.

[13] Nab M, van Vehmendahl R, Somers I, Schoon Y, Hesselink G (2021). Delayed emergency healthcare seeking behaviour by Dutch emergency department visitors during the first COVID-19 wave: a mixed methods retrospective observational study. BMC Emerg Med.

[14] Lazzerini M, Barbi E, Apicella A, Marchetti F, Cardinale F, Trobia G (2020). Delayed access or provision of care in Italy resulting from fear of COVID-19. Lancet Child Adolesc Health.

[15] Macy ML, Huetteman P, Kan K (2020). Changes in primary care visits in the 24 weeks after COVID-19 stay-at-home orders relative to the comparable time period in 2019 in Metropolitan Chicago and Northern Illinois. J Prim Care Community Health.

[16] Whaley CM, Pera MF, Cantor J, Chang J, Velasco J, Hagg HK (2020). Changes in health services use among commercially insured US populations during the COVID-19 pandemic. JAMA Netw Open.

[17] Stephenson E, Butt DA, Gronsbell J, Ji C, O'Neill B, Crampton N (2021). Changes in the top 25 reasons for primary care visits during the COVID-19 pandemic in a high-COVID region of Canada. PLoS ONE.

[18] Sigurdsson EL, Blondal AB, Jonsson JS, Tomasdottir MO, Hrafnkelsson H, Linnet K (2020). How primary healthcare in Iceland swiftly changed its strategy in response to the COVID-19 pandemic. BMJ Open.

[19] Schäfer I, Hansen H, Menzel A, Eisele M, Tajdar D, Lühmann D (2021). The effect of COVID-19 pandemic and lockdown on consultation numbers, consultation reasons and performed services in primary care: results of a longitudinal observational study. BMC Fam Pract.

[20] Coma E, Mora N, Méndez L, Benítez M, Hermosilla E, Fàbregas M (2020). M. Primary care in the time of COVID-19: monitoring the effect of the pandemic and the lockdown measures on 34 quality of care indicators calculated for 288 primary care practices covering about 6 million people in Catalonia. BMC Fam Pract.

[21] Glazier RH, Green ME, Wu FC, Frymire E, Kopp A, Kiran T (2021). Shifts in office and virtual primary care during the early COVID-19 pandemic in Ontario. Canada CMAJ.

[22] Santé Publique France (2021). COVID-19: Point épidémiologique hebdomadaire du 07 janvier 2021.

[23] Le Minez S, Roux V. 2020: une hausse des décès inédite depuis 70 ans. Insee Première. n° 1847. Mars 2021. https://www.insee.fr/fr/statistiques/5347349 (2021).

[24] Tuppin P, Rudant J, Constantinou P (2017). Value of a national administrative database to guide public decisions: from the Système National d'Information Interrégimes de l'Assurance Maladie (SNIIRAM) to the Système National des Données de Santé (SNDS) in France. Rev Epidemiol Sante Publique.

[25] Rey G, Jougla E, Fouillet A, Hémon D (2009). Ecological association between a deprivation index and mortality in France over the period 1997–2001: variations with spatial scale, degree of urbanicity, age, gender and cause of death. BMC Public Health.

[26] Constantinou P, Tuppin P, Fagot-Campagna A, Gastaldi-Ménager C, Schellevis FG, Pelletier-Fleury N (2018). Two morbidity indices developed in a nationwide population permitted performant outcome-specific severity adjustment. J Clin Epidemiol.

[27] Wasserstein RL, Schirm AL, Lazar NA (2019). Moving to a World Beyond “p<0.05”. Am Stat.

[28] Weill A, Drouin J, Desplas D, Cuenot, F, Dray-Spira R, Zureik M. Usage des médicaments de ville en France durant l'épidémie de la Covid-19 - point de situation jusqu'au 25 avril 2021. EPI-PHARE. https://www.epiphare.fr/rapports-detudes-et-publications/covid-19-usage-des-medicaments-r

[29] Gabet A, Grave C, Tuppin P, Lesuffleur T, Guenancia C, Nguyen-Thanh V (2022). Nationwide Initiation of Cardiovascular Risk Treatments During the COVID-19 Pandemic in France: Women on a Slippery Slope?. Front Cardiovasc Med.

[30] Moynihan R, Johansson M, Maybee A, Lang E (2020). Légaré F Covid-19: an opportunity to reduce unnecessary healthcare. BMJ.

[31] Hawkes MT, Lee BE, Kanji JN, Zelyas N, Wong K, Barton M, Mukhi S, Robinson JL (2021). Seasonality of respiratory viruses at northern latitudes. JAMA Netw Open.

[32] Wathelet M, Duhem S, Vaiva G, Baubet T, Habran E, Veerapa E (2020). Factors associated with mental health disorders among university students in France confined during the COVID-19 pandemic. JAMA Netw Open.

[33] Alexander GC, Tajanlangit M, Heyward J, Mansour O, Qato DM, Stafford RS (2020). Use and content of primary care office-based vs telemedicine care visits during the COVID-19 pandemic in the US. JAMA Netw Open.

[34] Joy M, McGagh D, Jones N, Liyanage H, Sherlock J, Parimalanathan V (2020). Reorganisation of primary care for older adults during COVID-19: a cross-sectional database study in the UK. Br J Gen Pract.

[35] Saint-Lary O, Gautier S, Le Breton J, Gilberg S, Frappé P, Schuers M (2020). How GPs adapted their practices and organisations at the beginning of COVID-19 outbreak: a French national observational survey. BMJ Open.

[36] Bramer CA, Kimmins LM, Swanson R, Kuo J, Vranesich P, Jacques-Carroll LA (2020). Decline in child vaccination coverage during the COVID-19 pandemic - Michigan Care Improvement Registry, May 2016-May 2020. MMWR Morb Mortal Wkly Rep.

[37] Chandler-Jeanville S, Nohra RG, Loizeau V, Lartigue-Malgouyres C, Zintchem R, Naudin D, Rothan-Tondeur M (2021). Perceptions and experiences of the COVID-19 pandemic amongst frontline nurses and their relatives in France in six paradoxes: a qualitative study. Int J Environ Res Public Health.

[38] Vilches-Moraga A, Price A, Braude P, Pearce L, Short R, Verduri A (2020). Increased care at discharge from COVID-19: The association between pre-admission frailty and increased care needs after hospital discharge; a multicentre European observational cohort study. BMC Med.

[39] Simon E, Cottenet J, Mariet AS, Bechraoui-Quantin S, Rozenberg P, Gouyon JB (2021). Impact of the COVID-19 pandemic on preterm birth and stillbirth: a nationwide, population-based retrospective cohort study. Am J Obstet Gynecol.

[40] Baumann S, Gaucher L, Bourgueil Y, Saint-Lary O, Gautier S, Rousseau A (2021). Adaptation of independent midwives to the COVID-19 pandemic: A national descriptive survey. Midwifery.

[41] Falvey JR, Krafft C, Kornetti D (2020). The essential role of home- and community-based physical therapists during the COVID-19 pandemic. Phys Ther.

[42] Gandré C, Coldefy M (2020). Disparities in the use of general somatic care among individuals treated for severe mental disorders and the general population in France. Int J Environ Res Public Health.

[43] van Aert GJJ, van der Laan L, Boonman-de Winter LJM, Berende CAS, de Groot HGW, Boele van Hensbroek P (2021). Effect of the COVID-19 pandemic during the first lockdown in the Netherlands on the number of trauma-related admissions, trauma severity and treatment: the results of a retrospective cohort study in a level 2 trauma centre. BMJ Open.

